# Supplementary material for: Imaging Voltage in Complete Neuronal Networks Within Patterned Microislands Reveals Preferential Wiring of Excitatory Hippocampal Neurons
Source: Front Neurosci. 2021 May 13;15:643868. doi: 10.3389/fnins.2021.643868 (PMC8155642; doi:10.3389/fnins.2021.643868)
Supplement: Supplementary file 1 [file Image_1.pdf]

1.1 Supplementary Figures

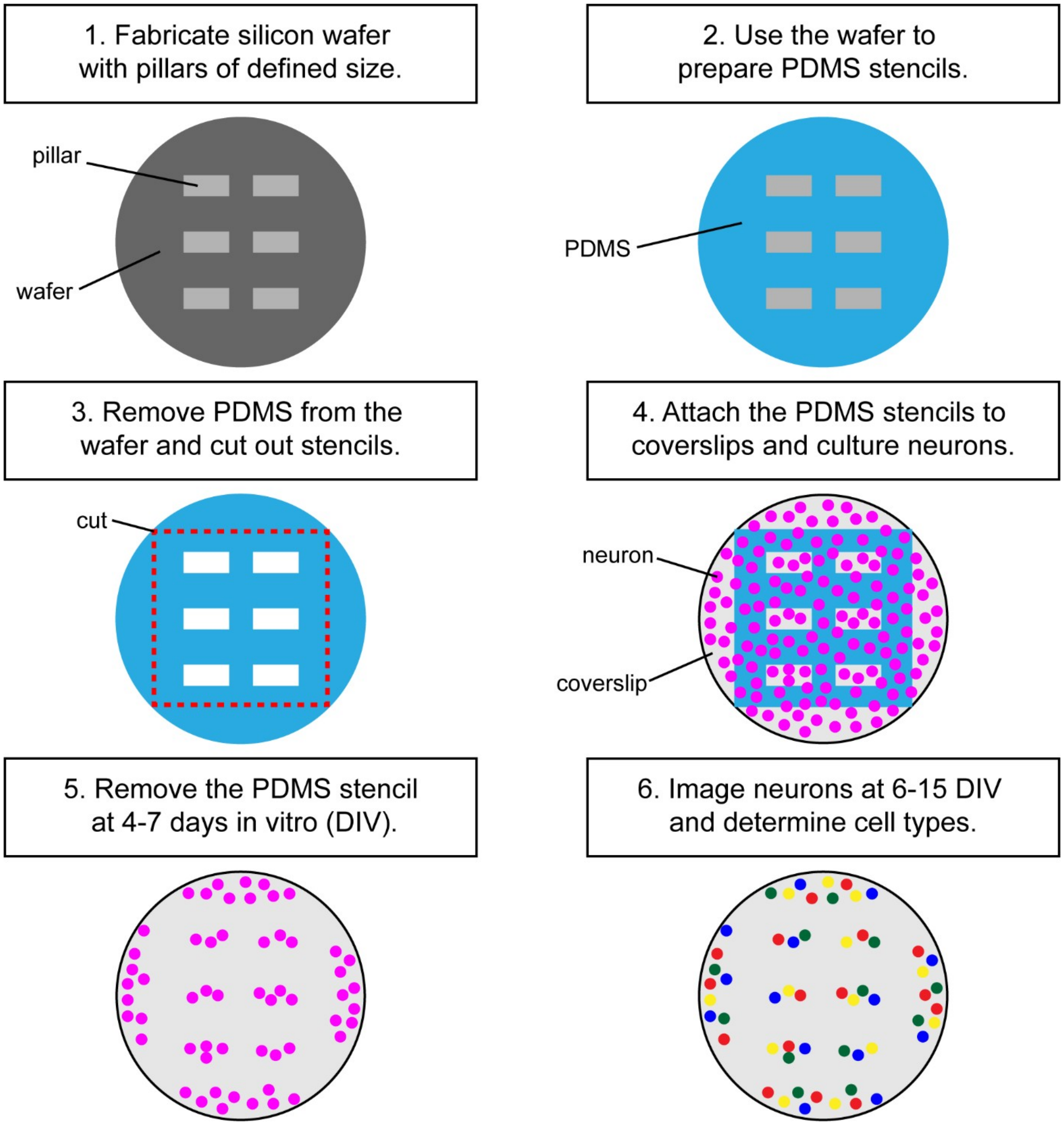

1.2 **Figure S1.** Microisland patterning workflow.

These diagrams are simplified for clarity. Typically, one Polydimethylsiloxane (PDMS) sheet yields 10 stencils containing 4 x 8 microislands. Typically, microislands contain 10-20 neurons.

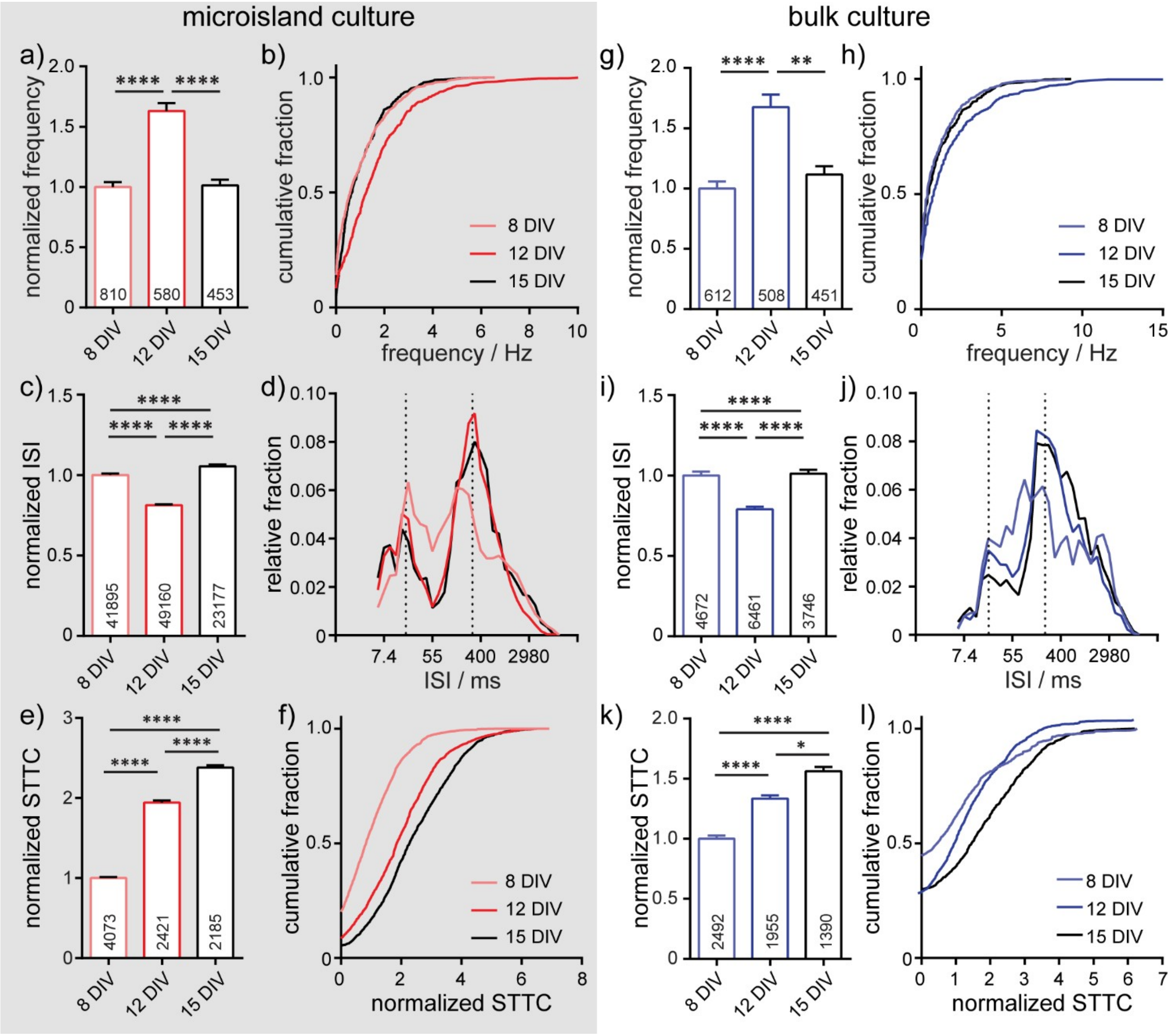

1.3 Figure S2. Comparison of neuronal activity in microisland and bulk culture.

Data in this figure are reproduced from Figure 3 in the main text (microisland culture) and from Figure 4 of Walker, *et al*. Optical spike detection and connectivity analysis with a far-red voltage-sensitive fluorophore reveals changes to network connectivity in development and disease. *Front. Neurosci.*, **2021**, DOI: 10.3389/fnins.2021.643859.

Characterization of neuronal activity in 650 × 120 µm microisland culture. Analyses of action potential frequency **(a,b)**, inter-spike interval, ISI, **(c,d)**, or spike timing tiling coefficient, STTC, **(e,f)** over developmental stages (8, 12, and 15 *days in vitro*; DIV) are summarized as a bar graph **(a,c,e)** and as a cumulative frequency plot **(b,f)**, or histogram **(d)**. Values on bar graphs indicate numbers of neurons **(a)**, pairs of spikes **(c)**, or pairs of neurons **(e)** analyzed for each condition.

Characterization of neuronal activity in bulk coverslip culture. Analyses of action potential frequency **(g,h)**, inter-spike interval, ISI, **(i,j)**, or spike timing tiling coefficient, STTC, **(k,l)** over developmental

stages (8, 12, and 15 *days in vitro*; DIV) are summarized as a bar graph **(g,i,k)** and as a cumulative frequency plot **(h,l)**, or histogram **(j)**. Values on bar graphs indicate numbers of neurons **(g)**, pairs of spikes **(i)**, or pairs of neurons **(l)** analyzed for each condition.

Data represent 4 biological replicates. Frequency, ISI, and STTC values are normalized to the 8 DIV value per biological replicate. Statistical tests are Kruskal-Wallis ANOVAs with multiple comparison tests to all groups. * = $p < 0.05$, ** = $p < 0.01$, *** = $p < 0.001$, **** = $p < 0.0001$.
